# Comparison Between Synchronized Intermittent Mandatory Ventilation (SIMV) and Adaptive Support Ventilation (ASV) on Patient Outcomes in Critically Ill Patients: A Systematic Review and Meta-Analysis

**DOI:** 10.7759/cureus.81165

**Published:** 2025-03-25

**Authors:** Ahmed M Abdelbaky, Wael G Elmasry, Ahmed H. Awad

**Affiliations:** 1 Critical Care Medicine, Mohammed Bin Rashid University of Medicine and Health Sciences (MBRU), Dubai, ARE; 2 Anaesthesia, Mohammed Bin Rashid University of Medicine and Health Sciences (MBRU), Dubai, ARE

**Keywords:** adaptive support ventilation, critical care, mechanical ventilation, medical intensive care unit (micu), synchronized intermittent mandatory ventilation

## Abstract

In critically ill patients, the choice of mechanical ventilation modes can have a significant impact on patient outcomes. The comparative efficacy of adaptive support ventilation (ASV) and synchronized intermittent mandatory ventilation (SIMV) remains debated in the literature. Therefore, this systematic review and meta-analysis aimed to investigate the impact of ASV compared to SIMV in critically ill patients. For this systematic review and meta-analysis, a thorough search was undertaken in PubMed, Web of Science, and Scopus. Primary outcomes included length of mechanical ventilation, ventilator days, and intensive care unit (ICU) length of stay (LOS), whereas secondary outcomes focused on physiological parameters (e.g., P-peak, hemodynamics, gas exchange). A total of 11 studies involving 627 patients were included. The results showed that ASV significantly reduced the length of mechanical ventilation (mean difference (MD): -0.80 days; 95% CI: -1.11 to -0.50; p<0.00001) and ventilator days (MD: -1.42 days; 95% CI: -1.83 to -1.01; p<0.00001) compared to SIMV. However, no differences were observed in ICU LOS (p=0.25), heart rate (p=0.17), minute volume (p=0.72), mean arterial pressure (p=0.26), PCO_2_ (p=0.97), PO_2_ (p=0.22), and respiratory rate (p=0.55). P-peak, however, was significantly higher in SIMV compared to ASV (MD: -2.16; 95% CI: -3.07 to -1.25; p<0.00001). The findings of the systematic review showed that ASV was associated with a shorter duration of mechanical ventilation and lower peak airway pressures than SIMV, which suggests its advantage in facilitating weaning and lung-protective ventilation. ASV may be preferable in ICU settings where reducing ventilation duration is critical. The findings of the present systematic review were limited by high heterogeneity and study quality variations. Therefore, further research is required to validate other non-significant outcomes.

## Introduction and background

Critically ill patients in intensive care facilities often present with respiratory failure or acute hypoxic respiratory insufficiency [1]. Mechanical ventilation is one of the most common therapeutic approaches for hospitalized patients in the intensive care unit (ICU) suffering from respiratory failure [2]. The purpose of mechanical ventilation is to ensure adequate oxygenation and carbon dioxide elimination. However, prolonged dependence on mechanical ventilation is a challenge, with the long-term outcomes of this dependence still incompletely understood [3]. There are several modes of mechanical ventilation, with each having certain advantages and disadvantages [2]. Synchronized intermittent mandatory (SIMV) and adaptive support ventilation (ASV) are two different modes of mechanical ventilation in ICU patients with different strategies that permit spontaneous breathing in patients, along with mandatory breaths by a ventilator in a coordinated method [4].

SIMV is a type of volume control mode of ventilation. In this mode of ventilation, the ventilator delivers a mandatory number of breaths with a fixed volume but, at the same time, allows spontaneous breaths in patients. Impulsive breaths are delivered when the airway pressure falls below the end-expiratory pressure [5]. SIMV is widely used for different levels of respiratory failure patients. It is also employed in the weaning process from mechanical ventilation. While effective, SIMV has notable drawbacks, including potential patient-ventilator asynchrony and the risk of over-assistance, which may lead to prolonged ventilation or ventilator-induced lung injury [6]. ASV mode is a kind of closed loop-pressure controlled ventilation mode. In ASV, respiratory rate and tidal volume are fixed by the machine in such a way that patients have to exert the least effort for breathing. The internal sensor of the ventilator could monitor body ventilation parameters effectively. They can also monitor the regulation of airflow speed-based requirements of patients and respiratory muscle mechanisms by nursing ventilation parameters. This monitoring can offer a more desirable coordination between the mechanical ventilation machine and the patient [2].

ASV is volume-targeted pressure support in spontaneously breathing patients [4]. Several studies have compared the effects of SIMV and ASV on clinical outcomes of critically ill patients. In a recent study by Basha et al., the ASV mode of ventilation has shown promising results in reducing the duration of ventilation and ICU stay [7]. Similarly, a previous systematic review and meta-analysis in which different ventilation modes were compared for patient outcomes found no difference between ASV and SIMV in the duration of hospital stay, duration of ICU stay, and mechanical ventilation [1]. Currently, there is no consensus on the best mode of mechanical ventilation for critical patients. Previously, no systematic review and meta-analysis has been conducted to investigate the comparative effectiveness of ASV and SIMV on patient outcomes in critically ill patients, leading to uncertainty in clinical decision-making. Furthermore, as there is a growing trend toward automation in ventilatory support, the comparison between these two ventilatory modes becomes even more crucial.

Therefore, this systematic review and meta-analysis was planned to evaluate the evidence about the effectiveness of SIMV and ASV in critically ill patients. The findings of the systematic review will help clinicians decide on the best approach for modes of mechanical ventilation in critical care.

## Review

Methodology 

This systematic review and meta-analysis followed published guidelines of the Cochrane Handbook for Systematic Reviews of Interventions [8]. The protocol for this systematic review and meta-analysis was registered on the International Prospective Register of Systematic Reviews (PROSPERO), with registration number CRD42024506813. The systematic review also followed the Preferred Reporting Items for Systematic Reviews and Meta-Analyses (PRISMA) guidelines [9]. The PICO framework for this systemic review and meta-analysis was as follows: patients (P) critically ill patients; intervention (I) ASV; control (C) SIMV; and outcomes (O) length of mechanical ventilation, number of ventilator days, ICU length of stay (LOS), heart rate, minute volume, mean arterial pressure, PCO_2_, PO_2_, P-peak, respiratory rate, and compliance.

Search Strategy and Data Sources

A thorough search was undertaken in key databases such as PubMed, Web of Science, and Scopus to identify relevant studies that compare ASV with SIMV in critically ill patients. Keywords used in the database search included a combination of “intensive care units,” “critical care,” “SIMV,” “ASV,” and “adaptive support ventilation.” The detail of further keywords is provided in Appendix 1. The search was carried out by combining keywords with boolean operators “OR” and “AND.” Furthermore, Google Scholar was searched to expand the number of potential studies. The inclusion of systematic review and meta-analysis was defined as follows: studies (1) comparing ASV and SIMV modes of ventilation, (2) involving critically ill subjects, and (3) publications available in English. The search was not limited based on study design or duration in order to identify a maximum number of studies from the literature. 

Data Extraction

The database search results were transferred to the reference manager (EndNote) to merge all references in a single file. The final file was transferred to Rayyan (Rayyan Systems Inc., Cambridge, MA), a software designed for study screening for systematic reviews [10]. In the first step, duplicates were removed. The remaining records were screened by two independent reviewers (AH, AB). To ensure that there is no bias in the screening process, the blind feature was turned on in Rayyan. Both reviewers first screened results based on their titles and abstracts. After finalizing the selection process, the blind was turned off and decisions by both reviewers were compared. Conflicts were resolved by mutual discussion; however, in case of any pending disagreements, a third reviewer (QA) was involved. After finalizing the included studies, data related to demographics and study outcomes were extracted in an Excel file.

Risk of Bias (ROB) Assessment

The ROB assessment also involved two reviewers (AH and AB). For randomized controlled trials, ROB 2 in Review Manager (RevMan; version 5.3, Cochrane Collaboration, London, UK) software was used. ROB was assessed in seven domains, including selection bias, performance bias, detection bias, attrition bias, reporting bias, and other biases. For crossover studies, ROB2 for crossover studies (developed by Cochrane) was used. ROB for crossover studies was assessed in five domains [11]. For cross-sectional studies, the Newcastle Ottawa Scale (NOS) was used for ROB involving three main domains (selection, comparability, and outcome).

Data Analysis

Meta-analysis was carried out by the DerSimonian and Laird method [12]. A random-effects model was chosen to account for the expected variability in the effects across the included studies, as the studies were conducted in different clinical settings, with varying patient populations, interventions, and methodologies. This approach allows for a more generalized estimate of the mean difference (MD) for continuous outcomes, along with corresponding 95% confidence intervals (CIs). The random-effects model assumes that the true effect size may vary between studies, which is typical in systematic reviews with heterogeneous study designs and populations. Heterogeneity was assessed using the I² statistic, with values below 25% suggesting low heterogeneity, 26%-50% as moderate, 51%-75% as substantial, and above 75% as high heterogeneity. A P-value of below 0.05 was considered statistically significant.

Results

Included Studies

A total of 727 studies were identified from various databases (PubMed=204, Web of Science=177, Scopus=278, Google Scholar=68). After removing 331 duplicates, 396 records were included in the screening process. Based on titles and abstracts of records, 364 records were excluded. In full-length screening, 32 articles were included. Only 11 articles met the inclusion criteria and were included in the systematic review.

Flow Diagram

**Figure 1:**
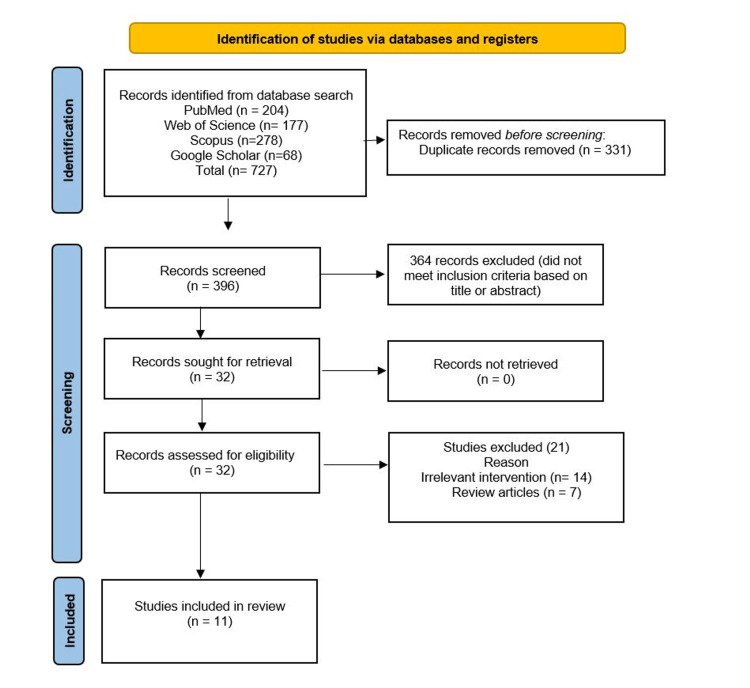
PRISMA flow diagram of the systematic review PRISMA: Preferred Reporting Items for Systematic Reviews and Meta-Analyses

Figure 1 shows the PRISMA flow diagram.

Characteristics of the Included Studies

Table 1 summarizes the characteristics of the included studies, comparing ASV and SIMV. A total of 627 patients were included in the systematic review, with 317 in ASV and 320 in the SIMV group. The majority of the studies were randomized studies (n=8), followed by cross-over studies (n=2) and one cross-sectional study. The mean age of participants varies from 32 to 71 years, with an overall average of around 52 years. The average number of female participants was 42.9 in the ASV and 42.0% in the SIMV group.

**Table 1:** Characteristics of the included studies ARDS: Acute respiratory distress syndrome; RCT: Randomized controlled trial; ICU: Intensive care unit

Authors	Year	Study Design	Participants			Age		Female n (%)		Patients
			Total	ASV	SIMV	ASV	SIMV	ASV	SIMV	
Yazdannik et al. [13]	2016	RCT	64	32	32	57.9±5.6	58.3±5.6	46.8	65.6	Coronary artery bypass graft surgery
Aghadavoudi et al. [14]	2012	RCT	81	41	40	57.9±8.9	59.8±12.7	34.1	42.5	Fast-track cardiac surgery
Zhang et al. [15]	2022	RCT	100	50	50	45.9±7.6	45.2±8.2	24	23	ARDS
Alikiaii et al. [4]	2022	RCT	24	12	12	59.7±16.9	47.8±20.6	50	27	ARDS
Celli et al. [16]	2014	RCT	20	10	10	58±5	56±3	10	10	Orthotopic liver transplantation
Haq et al. [17]	2023	RCT	100	50	50	36.7±10.0	35.8±10.8	44	40	ICU
Tassaux et al. [18]	2002	Prospective, crossover	10	10	10	71±9.6	71±9.6	60	60	Acute respiratory failure
Ghodrati et al. [2]	2016	Crossover study	60	30	30	54±6	54±6	47	47	Neurosurgical ICU
Alay et al. [19]	2023	Prospective randomized	60	30	30	52.5±18.9	59.7±11.9	50	30	Respiratory failure
Jabbari et al. [20]	2019	Cross-sectional case-control	68	32	36	58±5	56±6	56.2	47.2	Cardiac surgery
Doneria et al. [21]	2017	Prospective randomized	40	20	20	32±7	35±13	50	70	Intermittent positive pressure ventilation (IPPV)

ROB Assessment

**Figure 2:**
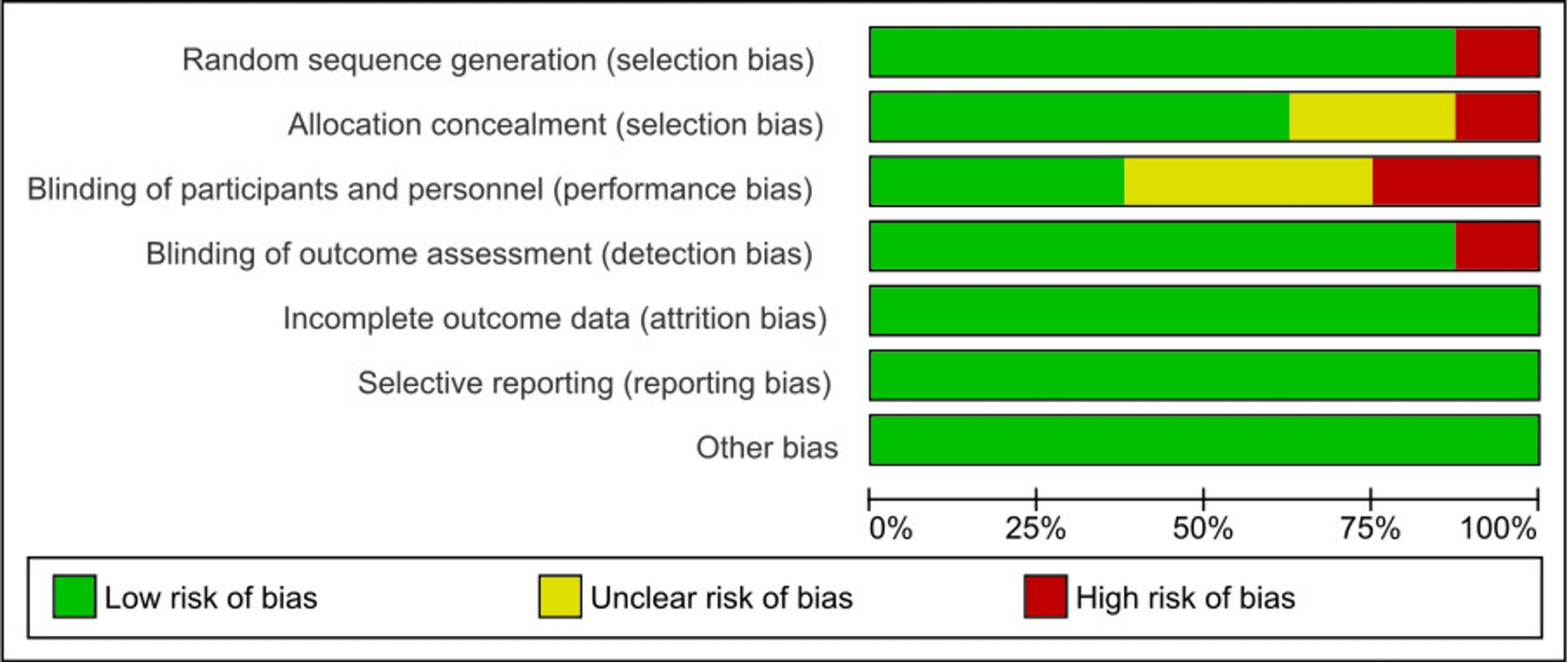
Risk of bias graph

**Figure 3:**
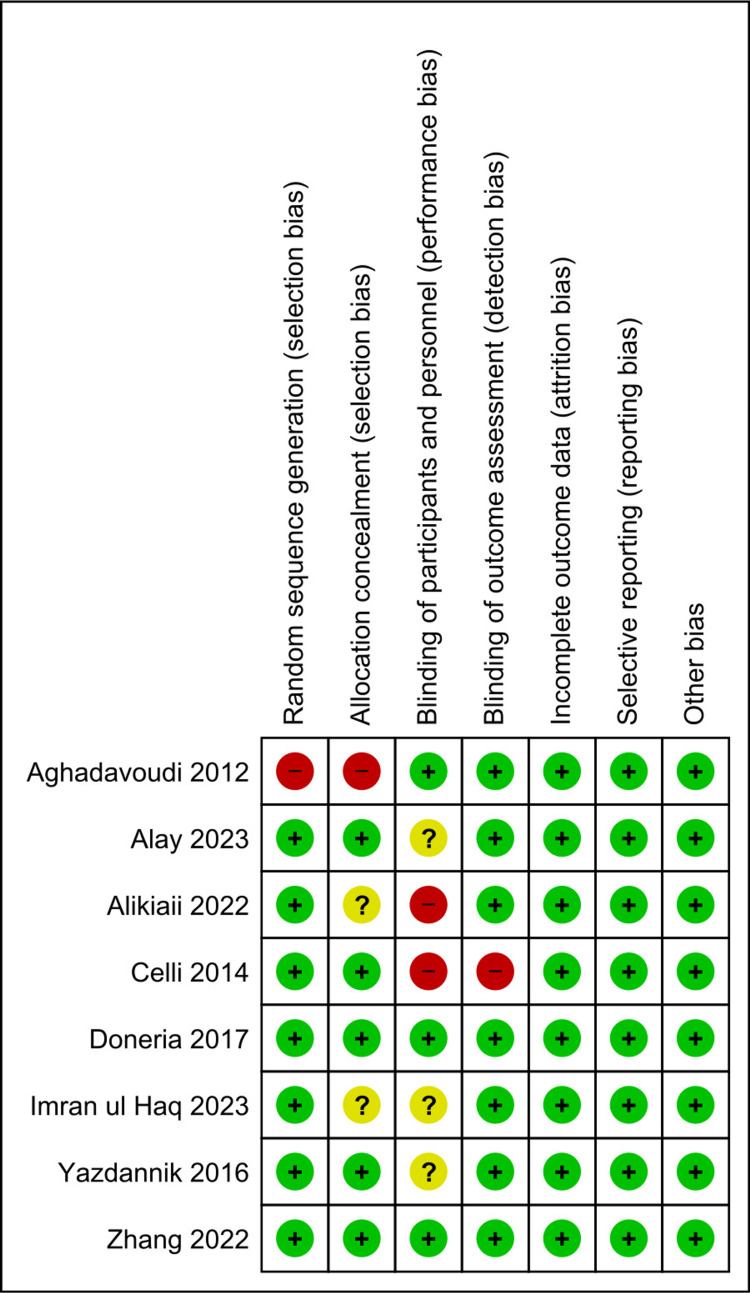
Risk of bias summary Yazdannik et al. [13], Aghadavoudi et al. [14], Zhang et al. [15], Alikiaii et al. [4], Celli et al. [16], Haq et al. [17], Alay et al. [19], Doneria et al. [21]

Figures 2-3 show the ROB graph and summary, respectively. ROB 2 was used to assess ROB in eight studies [4,13-17,19,21]. Selection bias was only high in one study [14], whereas, in all other studies, it was low. Similarly, allocation concealment-related bias was high in one study [14] and unclear in two studies [4,17]. Performance bias was high in two studies [4,16] and unclear in three studies [13,17,19]. Only Celli et al.'s [16] study had a high detection bias. No bias was found in other domains.

**Figure 4:**
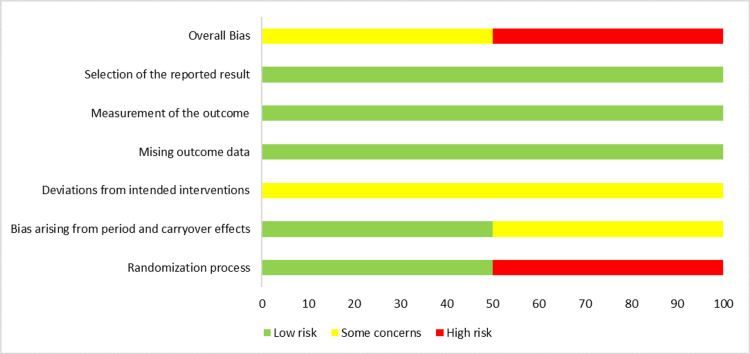
Risk of bias (ROB) 2 graph for cross-over studies in the systematic review

**Figure 5:**
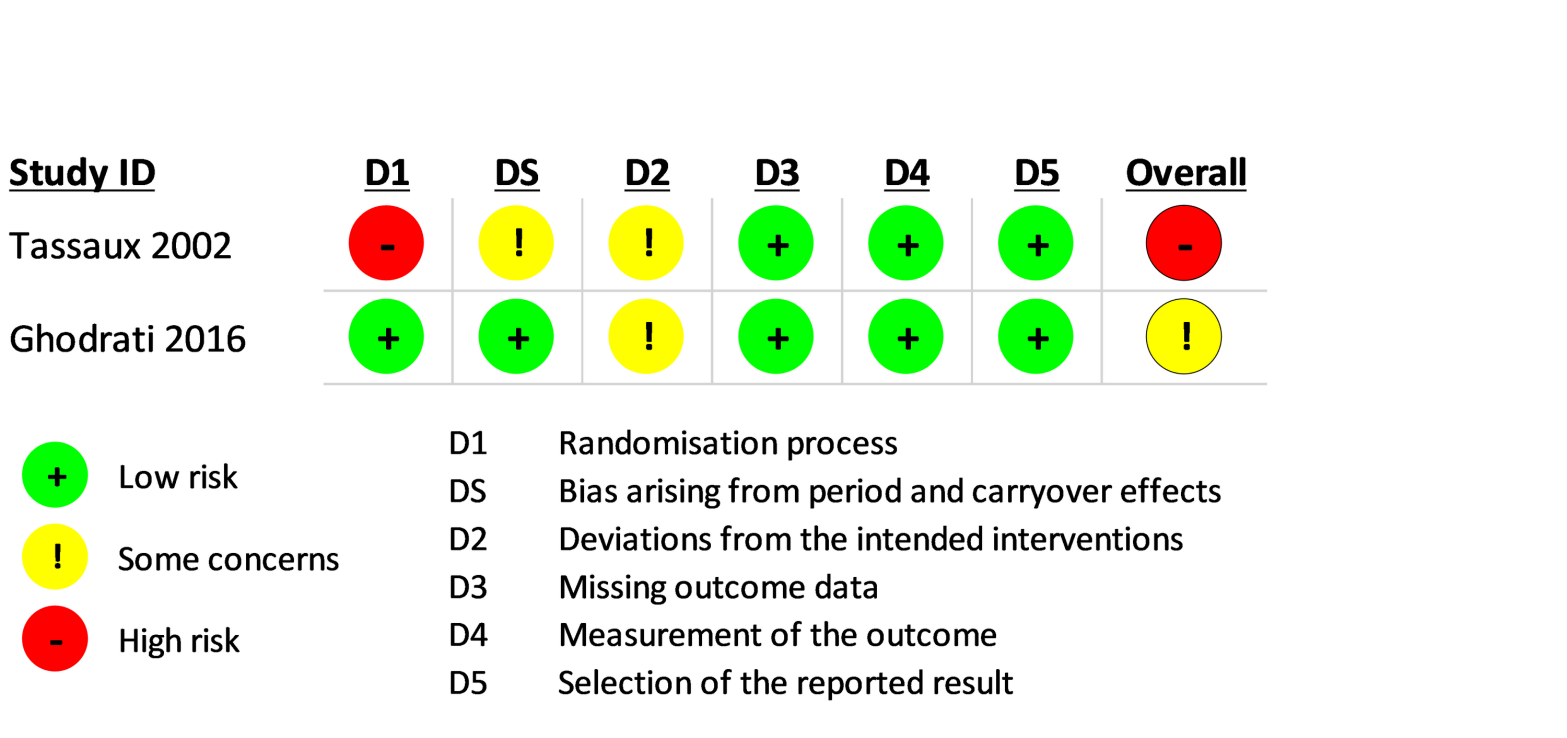
Risk of bias (ROB) 2 summary for cross-over studies in the systematic review Tassaux et al. [18], Ghodrati et al. [2]

ROB 2 for cross-over studies was used to assess bias in only two studies [2,18]. ROB was high in the randomization domain in Tassaux et al. [18], whereas, in the domain pertaining to bias related to period and carryover effects, there were some concerns. Similarly, there were also some concerns related to D2, focusing on intended interventions in Tassaux et al. [18]. Overall, ROB was high in Tassaux et al. [18]. On the other hand, Ghodrati et al. [2] showed some concerns in D2, related to deviation from intended interventions. Overall the ROB had some concerns in Ghodrati et al. [2] (Figures 4-5).

Table 2 shows the ROB measured with the Newcastle Ottawa Scale (NOS) in the cross-sectional study by Jabbari et al. [20]. ROB was low in all domains, including selection, comparability, and outcome.

**Table 2:** Risk of bias measured with the Newcastle Ottawa Scale (NOS) Jabbari et al. [20]

Study	Selection				Comparability		Outcome			Total quality score
	Representativeness of the exposed cohort	Selection of the non-exposed cohort	Ascertainment of exposure	Demonstration that outcome of interest was not present at the start of study	Controls for the most important risk factors	Controls for other risk factors	Assessment of outcome	Was follow-up long enough for outcomes to occur	Adequacy of follow-up of cohorts	
Jabbari et al. 2019	1	1	1	1	1	1	1	1	1	9

Length of Mechanical Ventilation

**Figure 6:**
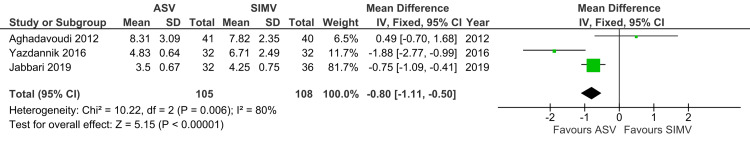
Forest plot showing the length of mechanical ventilation ASV: Adaptive support ventilation; SIMV: Synchronized intermittent mandatory ventilation Aghadavoudi et al. [14], Yazdannik et al. [13], Jabbari et al. [20]

Only three studies reporting the length of mechanical ventilation were included in the analysis. The length of mechanical ventilation was significantly lower in ASV compared to the SIMV group (MD: -0.80; 95% CI: -1.11 to -0.50; p<0.00001; I^2^=80%) (Figure 6).

Number of Ventilator Days

**Figure 7:**
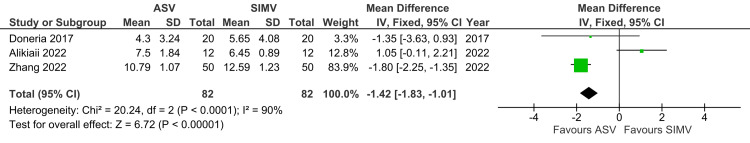
Forest plot showing the number of ventilator days ASV: Adaptive support ventilation; SIMV: Synchronized intermittent mandatory ventilation Doneria et al. [21], Alikiaii et al. [4], Zhang et al. [15]

Number of ventilator days was significantly lower in ASV compared to the SIMV group (MD: -1.42; 95% CI: -1.83 to -1.01; p<0.00001, I2=90%) (Figure 7).

ICU LOS

**Figure 8:**
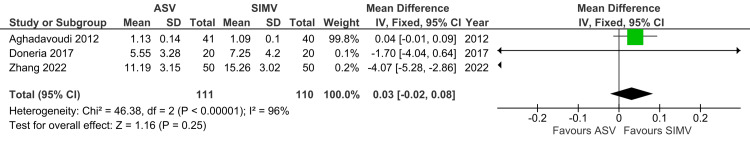
Forest plot showing ICU LOS ICU LOS: Intensive care unit (ICU) length of stay (LOS) Aghadavoudi et al. [14], Doneria et al. [21], Zhang et al. [15]

A total of three studies reported ICU LOS and were included in the analysis. ICU LOS was not significantly different between the ASV and SIMV groups (MD: 0.03; 95% CI: -0.02 to 0.08; p=0.25, I^2^=96%) (Figure 8).

Heart Rate

**Figure 9:**
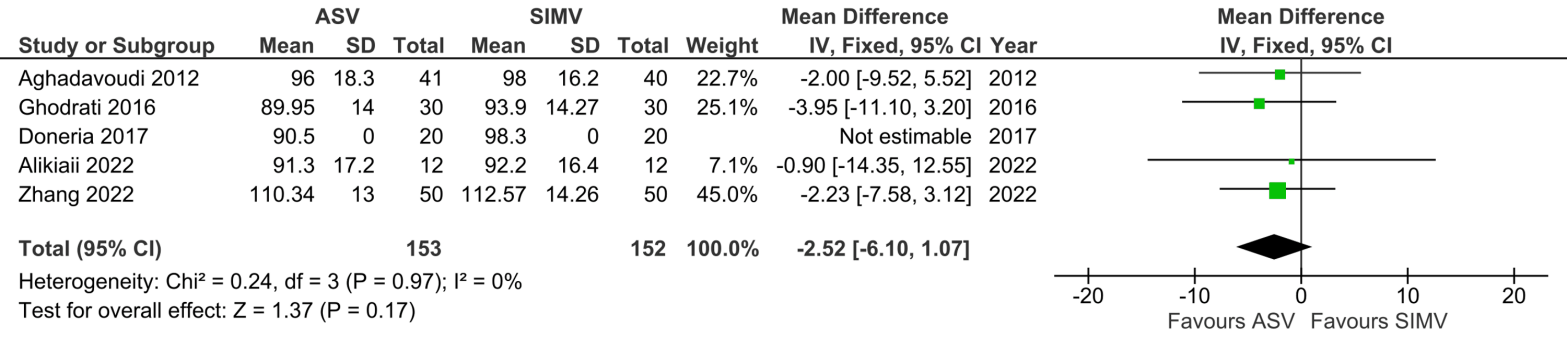
Forest plot showing the heart rate ASV: Adaptive support ventilation; SIMV; Synchronized intermittent mandatory ventilation Aghadavoudi et al. [14], Ghodrati et al. [2], Doneria et al. [21], Alikiaii et al. [4], Zhang et al. [15]

Five studies reporting heart rate outcomes were included in the analysis. Heart rate was not significantly different between the ASV and SIMV groups (MD: -2.52; 95% CI: -6.10 to 1.07; p=0.17). The heterogeneity was low (I^2^=0%) (Figure 9).

Minute Volume

**Figure 10:**
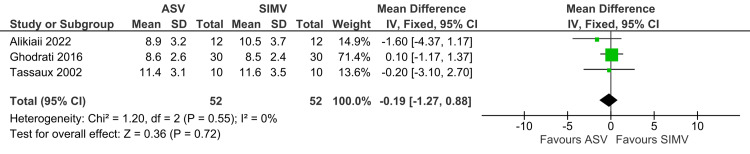
Forest plot showing minute volume ASV: Adaptive support ventilation; SIMV: Synchronized intermittent mandatory ventilation Ghodrati et al. [2], Alikiaii et al. [4], Tassaux et al. [18]

Minute volume was not significantly different between the ASV and SIMV groups (MD: -0.19; 95% CI: -1.27 to 0.88; p=0.72; I^2^=0%) (Figure 10).

Mean Atrial Pressure

**Figure 11:**
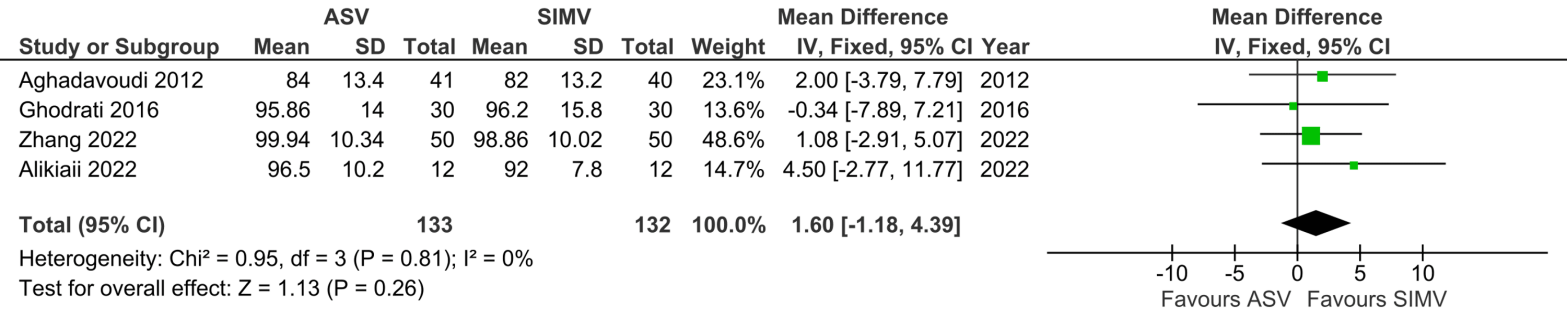
Forest plot showing MAP ASV: Adaptive support ventilation; SIMV: Synchronized intermittent mandatory ventilation Aghadavoudi et al. [14], Ghodrati et al. [2], Alikiaii et al. [5], Zhang et al. [15]

A total of four studies reported MAP and were included in the analysis. MAP was not significantly different between the ASV and SIMV groups (MD: 1.60; 95% CI: -1.18 to 4.39; p=0.26; I^2^=0%) (Figure 11).

PCO_2_

**Figure 12:**
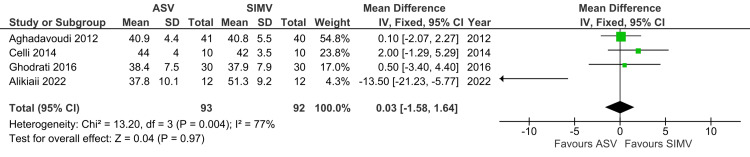
Forest plot showing PCO2 ASV: Adaptive support ventilation; SIMV: Synchronized intermittent mandatory ventilation Aghadavoudi et al. [14], Celli et al. [16], Ghodrati et al. [2], Alikiaii et al. [4]

A total of four studies reported PCO2 and were included in the analysis. PCO2 was not significantly different between the ASV and SIMV groups (MD: 0.03; 95% CI: -1.58 to 1.64; p=0.97; I^2^=77%) (Figure 12).

PO_2_

**Figure 13:**
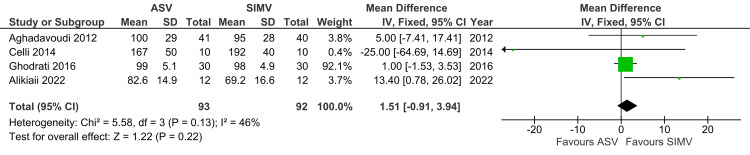
Forest plot showing PO2 ASV: Adaptive support ventilation; SIMV: Synchronized intermittent mandatory ventilation Aghadavoudi et al. [14], Celli et al. [16], Ghodrati et al. [2], Alikiaii et al. [4]

A total of four studies reported PO2 and were included in the analysis. PO2 was not significantly different between the ASV and SIMV groups (MD: 1.51; 95% CI: -0.91 to 3.94; p=0.22; I^2^=46%) (Figure 13).

P-Peak

**Figure 14:**
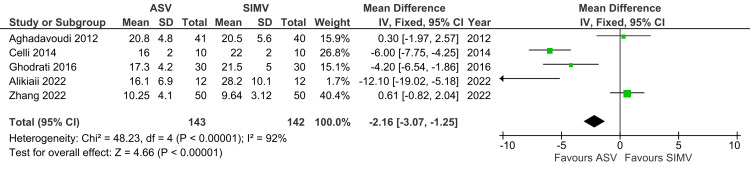
Forest plot showing P-peak ASV: Adaptive support ventilation; SIMV: Synchronized intermittent mandatory ventilation Aghadavoudi et al. [14], Celli et al. [16], Ghodrati et al. [2], Alikiaii et al. [4], Zhang et al. [15]

A total of five studies reported a number of P-peak and were included in the analysis. P-peak was significantly lower in ASV compared to the SIMV group (MD: -2.16; 95% CI: -3.07 to -1.25; p<0.00001; I^2^=92%) (Figure 14).

Respiratory Rate

**Figure 15:**
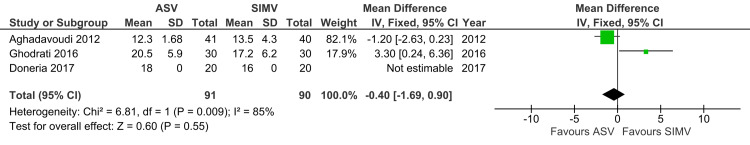
Forest plot showing the respiratory rate ASV: Adaptive support ventilation; SIMV: Synchronized intermittent mandatory ventilation Aghadavoudi et al. [14], Ghodrati et al. [2], Doneria et al. [21]

A total of three studies reported respiratory rate and were included in the analysis. Respiratory rate was not significantly different between the ASV and SIMV groups (MD: -0.40; 95% CI: -1.69 to 0.90; p=0.55; I^2^=85%) (Figure 15).

Compliance

**Figure 16:**
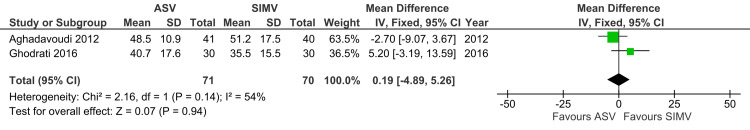
Forest plot showing compliance ASV: Adaptive support ventilation; SIMV: Synchronized intermittent mandatory ventilation Aghadavoudi et al. [14], Ghodrati et al. [2]

A total of two studies reported compliance and were included in the analysis. Compliance was not significantly different between the ASV and SIMV groups (MD: 0.19; 95% CI: -4.89 to 5.26; p=0.94; I^2^=54%) (Figure 16).

Discussion 

This systematic review and meta-analysis evaluated the impact of ASV versus SIMV on clinical outcomes in critically ill patients. The findings from 11 studies, including 627 patients, revealed that ASV significantly reduced the length of mechanical ventilation and number of ventilator days compared to SIMV. Furthermore, ASV was associated with lower peak airway pressures. However, no significant differences were observed in ICU LOS, hemodynamic parameters (heart rate, mean arterial pressure), gas exchange metrics (PCO₂, PO₂), respiratory rate, or lung compliance. The reason for no significant difference in ICU LOS in the present systematic review was that it is influenced by multiple factors, including comorbidities, hospital policies, and post-extubation care, which were not standardized across studies. Furthermore, differences in clinician-adjusted ventilator settings could have influenced ASV and SIMV effectiveness, leading to variability in outcomes.

To the best of our knowledge, this is the first systematic review that compared ASV with SIMV in critically ill patients. Previously, some systematic reviews have investigated different mechanical ventilations, including SIMV and ASV [1,22]. For example, a systematic review and meta-analysis by Kampolis et al. compared the efficacy of different ventilation modes on weaning patients of invasive mechanical ventilation, including ASV in critically ill patients [22]. Their findings showed that proportional assist ventilation (PAV) demonstrated better outcomes compared to other ventilation modes. However, they did not include SIMV in their analysis. The most comparable systematic review and meta-analysis to the present study was done by Wu et al., which included seven ventilation modes. Their results included 28 RCTs. They reported that patient outcomes such as ICU stay, and mechanical ventilation duration were not significantly different in the SIMV group compared to other ventilation modes [1]. A limitation of their systematic review is that they did not compare ASV and SIMV.

Previous studies that have compared ASV with other ventilation modes have reported that ASV leads to lower ventilation time. For example, Kirkali et al. reported that, in the ASV group, the duration of mechanical ventilation was significantly lower compared to pressure assist/control ventilation (p=0.003) [23]. Similarly, a systematic review by Rose et al. that compared automated and non-automated ventilation modes reported that automated modes such as ASV lowered the duration of mechanical ventilation compared to non-automated ventilation modes [24]. Regarding lower P-peak observed in the present systematic review in ASV groups aligns with previous studies. For example, Fathi et al., in their study, reported fewer P-peak observed in the ASV group compared to PSV in patients undergoing coronary artery bypass graft surgery [25]. The reduction in mechanical ventilation duration and P-peak with ASV aligns with its closed-loop design, which dynamically adjusts inspiratory pressure and respiratory rate based on real-time measurements of patient effort and lung mechanics. Furthermore, as ASV modes have lower peak airway pressures, which results in less work of breathing, it can explain the findings related to lower ventilation time that is observed in ASV modes [26].

This is unlike SIMV in which fixed mandatory breaths are delivered regardless of patient demand. This way ASV minimizes ventilator-patient asynchrony, a common issue linked to prolonged weaning and diaphragmatic atrophy [27]. This adaptive approach likely explains the shorter ventilator days, as ASV tailors support to metabolic needs, preventing over-assistance, which delays weaning, or under-assistance, which increases the work of breathing [28]. The lower P-peak observed with ASV is clinically significant, as elevated airway pressures are a well-established risk factor for ventilator-induced lung injury. For example, a study by Dai et al., which included both animal and human participants, reported that ASV reduces the risk of ventilator-induced lung injury [29]. They further reported that ASV has breathing patterns that align with lung protective strategies.

Strengths and Limitations

The main strength of this systematic review and meta-analysis is that it is the first meta-analysis to date to compare ASV with SIMV in critically ill patients. Furthermore, a robust systematic search was carried out with high-quality databases to identify relevant studies. Another strength of this systematic review and meta-analysis is that it did not limit the inclusion of studies based on study design or duration, which led to the inclusion of more studies in the analysis. There are several limitations of this systematic review and meta-analysis as well, which should be considered while interpreting the findings. Firstly, we were able to perform study-level meta-analysis. Secondly, we pooled the data from RCTs with cross-over and observational studies, which lowered the power of the analysis. Furthermore, studies did not report uniform outcomes across all studies. This also led to a reduced number of studies included in the meta-analysis. Due to the lower number of studies reporting each outcome, it was not possible to perform a subgroup analysis. There was also a high risk of bias in one crossover study. Furthermore, there was high heterogeneity, which could have been introduced due to variability in study protocols and patient populations. Most of the studies were unblinded, which could have introduced bias in favor of ASV due to their automated mode. Another limitation was that most studies focused on short-term outcomes, and data on long-term outcomes, such as mortality, were missing from the studies. Another limitation of the present systematic review was the limited number of participants included in the studies, which may not allow generalizability of the findings. Furthermore, we did not perform publication bias for the included studies.

Implications for Practice and Research

The findings of this systematic review and meta-analysis based on data from 627 patients showed that ASV leads to significantly lower length of mechanical ventilation and peak airway pressure compared to SIMV. However, there was no significant difference in other outcomes such as ICU LOS, heart rate, minute volume, MAP, PCO_2_, PO_2_, respiratory rate, and compliance. These findings show the clinical utility of ASV, especially in settings where reducing ventilator duration is critical (e.g., resource-limited ICUs, post-operative care). The lower P-peak with ASV supports its use in ARDS or chronic obstructive pulmonary disease (COPD) to mitigate barotrauma, though concomitant monitoring of plateau pressures remains essential. 

The findings also have implications for research. Currently, there is a paucity of research that has compared ASV with SIMV in critically ill patients; therefore, further research is necessary to compare ASV with SIMV. There is also a limited number of RCTs published on this topic. Therefore, there is a need for more high-quality evidence from RCTs to compare ASV with SIMV. Furthermore, a larger sample size should be aimed for in these RCTs, as currently most studies on this topic have a small sample size. Studies should also assess the long-term outcomes of ASV and SIMV exposure, such as mortality and hospital LOS.

## Conclusions

The findings of this systematic review and meta-analysis showed that ASV reduces mechanical ventilation duration and peak pressures compared to SIMV. However, no significant difference was observed in other patient outcomes including ICU LOS and hemodynamic parameters. However, there was high heterogeneity in the included studies. Furthermore, the findings of the systematic review highlight some limitations as well including small sample size included in studies, limited number of RCTs, and lack of long-term outcomes. Future studies should focus on large RCTs with long-term follow-ups to compare ASV with SIMV in critically ill patients. 

## Appendices

**Table 3:** Keyword combination used in the database search

Key Variable	Sub-terms	Search options	PubMed	Web of Science	Scopus
1. Ventilation	#1 synchronized intermittent mandatory ventilation	TI/AB	287	298	393
	#2 adaptive support ventilation	TI/AB	115	300	379
	#3 SIMV	TI/AB	321	265	442
	#4 ASV	TI/AB	6481	4885	6205
(((ASV[Title/Abstract]) OR (SIMV[Title/Abstract])) OR (adaptive support ventilation [Title/Abstract])) OR (synchronized intermittent mandatory ventilation[Title/Abstract])			6936	5470	7022
2. Critically Ill	#1 Critical Illness	Mesh	42,144		
	#2 Critical Illness	TI/AB	15,168	25,881	33,962
	#3 Critical Illnesses	TI/AB	889	25,881	33,962
	#4 Critically Ill	TI/AB	66,733	64,878	77,468
	#5 Intensive Care Units	Mesh	114,150		
	#6 Intensive Care Units	TI/AB	37,784	163,727	205,801
	#7 Intensive Care Unit	TI/AB	148,398	163,727	205,801
	#8 ICU	TI/AB	97,103	93,968	112,196
((((((("Critical Illness"[Mesh]) OR ("Intensive Care Units"[Mesh])) OR (Critical Illness[Title/Abstract])) OR (Critical Illnesses[Title/Abstract])) OR (Critically Ill[Title/Abstract])) OR (Intensive Care Units[Title/Abstract])) OR (Intensive Care Unit[Title/Abstract])) OR (ICU[Title/Abstract])			295,550	262,085	320,519
3. (((((((("Critical Illness"[Mesh]) OR ("Intensive Care Units"[Mesh])) OR (Critical Illness[Title/Abstract])) OR (Critical Illnesses[Title/Abstract])) OR (Critically Ill[Title/Abstract])) OR (Intensive Care Units[Title/Abstract])) OR (Intensive Care Unit[Title/Abstract])) OR (ICU[Title/Abstract])) AND ((((ASV[Title/Abstract]) OR (SIMV[Title/Abstract])) OR (adaptive support ventilation [Title/Abstract])) OR (synchronized intermittent mandatory ventilation[Title/Abstract]))			204	177	278
